# Enhanced effect of seasonal malaria chemoprevention when coupled with nutrients supplementation for preventing malaria in children under 5 years old in Burkina Faso: a randomized open label trial

**DOI:** 10.1186/s12936-023-04745-6

**Published:** 2023-10-18

**Authors:** Paul Sondo, Bérenger Kaboré, Toussaint Rouamba, Eulalie Compaoré, Yssimini Nadège Guillène Tibiri, Hyacinthe Abd-El Latif Faïçal Kaboré, Karim Derra, Marc Christian Tahita, Hamidou Ilboudo, Gauthier Tougri, Ismaïla Bouda, Tikanou Dakyo, Hyacinthe Kafando, Florence Ouédraogo, Eli Rouamba, So-vii Franck Hien, Adama Kazienga, Cheick Saïd Compaoré, Estelle Bambara, Macaire Nana, Prabin Dahal, Franck Garanet, William Kaboré, Thierry Léfèvre, Philippe Guerin, Halidou Tinto

**Affiliations:** 1 Institut de Recherche en Sciences de La Santé/Clinical Research Unit of Nanoro (IRSS-URCN), Nanoro, Burkina Faso; 2 Ministry of Health of Burkina Faso/Ouagadougou, Ouagadougou, Burkina Faso; 3 Malaria Consortium, Ouagadougou, Burkina Faso; 4 Infectious Diseases Data Observatory (IDDO)-WorldWide Antimalarial Resistance Network (WWARN), Oxford, UK; 5 Maladies Infectieuses et Vecteurs: Ecologie, Génétique, Evolution et Contrôle (MIVEGEC), Université de Montpellier, Institut de Recherche Pour le Développement (IRD), Centre National de la Recherche Scientifique (CNRS), Montpellier, France

**Keywords:** Malaria, Malnutrition, Seasonal chemo-prevention, PlumpyDoz^™^, Vitamin A, Zinc

## Abstract

**Background:**

In rural African settings, most of the children under the coverage of Seasonal Malaria Chemoprevention (SMC) are also undernourished at the time of SMC delivery, justifying the need for packaging malarial and nutritional interventions. This study aimed at assessing the impact of SMC by coupling the intervention with nutrients supplementation for preventing malaria in children less than 5 years old in Burkina Faso.

**Methods:**

A randomized trial was carried out between July 2020 and June 2021 in the health district of Nanoro, Burkina Faso. Children (n = 1059) under SMC coverage were randomly assigned to one of the three study arms SMC + Vitamin A (SMC-A, n = 353) or SMC + Vitamin A + Zinc (SMC-AZc, n = 353) or SMC + Vitamin A + PlumpyDoz(tm) (SMC-APd, n = 353)-a medium quantity—lipid-based nutrient supplement (MQ-LNS). Children were followed up for one year that included an active follow-up period of 6 months with scheduled monthly home visits followed by 6 months passive follow-up. At each visit, capillary blood sample was collected for malaria diagnosis by rapid diagnosis test (RDT).

**Results:**

Adding nutritional supplements to SMC had an effect on the incidence of malaria. A reduction of 23% (adjusted IRR = 0.77 (95%CI 0.61–0.97) in the odds of having uncomplicated malaria in SMC-APd arm but not with SMC-AZc arm adjusted IRR = 0.82 (95%CI 0.65–1.04) compare to control arm was observed. A reduction of 52%, adjusted IRR = 0.48 (95%CI 0.23–0.98) in the odds of having severe malaria was observed in SMC-APd arm compared to control arm. Besides the effect on malaria, this combined strategy had an effect on all-cause morbidity. More specifically, a reduction of morbidity odds of 24%, adjusted IRR = 0.76 (95%CI 0.60–0.94) in SMC-APd arm compared to control arm was observed. Unlike clinical episodes, no effect of nutrient supplementation on cross sectional asymptomatic infections was observed.

**Conclusion:**

Adding nutritional supplements to SMC significantly increases the impact of this intervention for preventing children from malaria and other childhood infections.

*Trial registration*: NCT04238845.

## Background

Malaria remains a life-threatening infectious disease causing about 619,000 deaths annually in the world, with children under 5 years of age accounting for about 80% of all malaria related deaths [1]. In order to reduce malaria burden in children, seasonal malaria chemoprevention (SMC) was recommended since 2012 by the World Health Organization (WHO) in areas where malaria transmission is seasonal [2]. SMC is an effective malaria prevention strategy consisting of monthly administration of therapeutic courses of amodiaquine and sulfadoxine-pyrimethamine (AQSP) in children (aged 3 to 59 months) during the high transmission period through a door-to-door delivery strategy. In 2021, up to 15 countries with intermittent malaria transmission implemented SMC at scale. In Burkina Faso, SMC started in 2014 with 7 health districts and was scaled-up nationally in 2019.

Although SMC intervention has been shown to be very effective [3, 4], the full potential of this intervention may have not been achieved after 10 years of implementation in Bukina Faso. This necessitated innovative approaches to tailor the SMC programme to the local context. This included extension to children aged older than 5 years [5, 6] as well as the number of SMC cycles depending on the length of the transmission season [7], and recently the extension to others parts of Africa with no more geographic restrictions [8]. Such adaptation of SMC to the local context is crucial, offering the opportunity to address local specificities that can influence the effectiveness of the intervention. For instance, in Burkina Faso, most of the children under the coverage of SMC are also undernourished at the time of SMC delivery [9]. Children under five are the most vulnerable group for both malaria and malnutrition and in terms of periodicity, these two major causes of infant morbidity and mortality peak during the rainy season which also corresponds to the period food shortage in the country. Although the relationship between malnutrition and susceptibility to malaria is not clearly established with controversial results (either positively [10–13], or negatively [14, 15] or with no effect [16, 17]), it is clear that malnutrition weakens the immune system of children and could also influence the drug pharmacokinetics [18]. Therefore, malnutrition could be seen as one of the potential factors that can negatively affect the effectiveness of SMC. Furthermore, children suffering from severe acute malnutrition are not eligible for SMC, justifying the integration of malnutritional status screening during SMC campaigns and this was effective since 2019 in Burkina Faso. More interestingly, treating micronutrient deficiencies has also been reported to have a protective effect against malaria [19, 20]. Medium Quantity Lipid-based Nutrient Supplement (MQ-LNS) specially designed to reduce the incidence of (severe) acute malnutrition, such as Plumpy’Doz, are currently available on the market [21]. In view of all these aspects, considering a strategy combining SMC with these nutritional supplements could be justified to improve the protective effect of SMC against malaria in children. A similar approach was previously assessed in Nigeria [22], but the evaluation was based on cross-sectional surveys. This study was one of the first randomized trials aiming at assessing the impact of SMC by coupling the intervention with nutritional supplementation for preventing malaria in children in Burkina Faso.

## Methods

### Study area

The study was carried out in a rural setting in the Central-western part of Burkina Faso, at the department of Soaw, one of the five departments of the health district of Nanoro. The study site included the coverage area of the health centre of Soaw (village of Soaw and village of Rakalo) and the coverage area of the health and social promotion centre of Zoetgomdé (village of Zoetgomdé and village of Kalwaka). According to the health and demographic surveillance system (HDSS) of Nanoro, the population in 2020 was estimated at 7293 inhabitants for the village of Soaw, 2179 inhabitant for the village Rakalo, 931 inhabitants for the village of Zoetgomdé and 2262 inhabitants for the village of Kalwaka [23]. Malaria transmission is seasonal with a high transmission peak during the rainy season, making SMC an excellent malaria prevention strategy in the area [24]. Almost all cases of malaria are caused by *Plasmodium falciparum*. The baseline assessment showed a high prevalence of child malnutrition during the rainy season in the area [9].

### Study design

This was a randomized open label trial carried out between July 2020 and June 2021. The study protocol was published previously [25]. Children were included on the basis of the following criteria: (i) children aged 6–59 months old and living under SMC coverage who received Vitamin A supplementation, (ii) permanent resident within the study area, (iii) ability to complete the 12 months follow-up period, and (iv) willingness of parents / guardians to participate to the study. Children were excluded when they were not covered by SMC or Vitamin A supplementation: (i) individual not under both interventions’ coverage, or in case of illness at the time of the enrolment, or known allergy to study drugs i.e. Vitamin A or Zinc or Plumpy’Doz™, or finally in case of inability to complete the study follow-up, or unwillingness of parents/guardian to participate to the study.

Included children were randomly assigned following a computer-generated randomization list to one of the three study arms SMC + Vitamin A (SMC-A) or SMC + Vitamin A + Zinc (SMC-AZc) or SMC + Vitamin A + PlumpyDoz™, (SMC-APd) a Medium Quantity Lipid-based Nutrient Supplement. Single dose of 200000UI of Vitamin A per child was administered within 3 weeks preceding the start of SMC campaign. One sachet of PlumpyDoz™ (Nutriset, Malaunay, France) was offered daily over a period of 3 months while 10 mg of elemental Zinc was administered daily in 6 days per week for the same period as for PlumpyDoz™ (3 months). The supply of nutritional supplements was done every two weeks and at each contact during monthly home visits. The first dose was administered under supervision by the field worker. Children were followed up for one year including an active follow-up period of 6 months with scheduled monthly home visits followed by a 6 months passive follow-up period i.e. no scheduled home visit during that period. Besides the monthly home visits, parents were advised to bring back the children to the health centres when they were sick. The research nurses team was posted at each health centre to collect all the study related information during participants’ visits.

### Participant’s assessment

At each visit, physical examination was performed. Body temperature was determined using a contactless thermometer and reported directly on degree Celsius. Direct interview was performed for assessing any occurrences of apparent and common symptoms regarding a pre-defined list of symptoms. Capillary blood sample was collected for malaria diagnosis by RDT and for haemoglobin measurement. Histidine Rich Protein-2 (HRP-2) RDT was used. Haemoglobin was measured using HemoCue® 201 + and Hb level was directly recorded in g/dL. Any participant requiring medical care detected during the home visit was immediately referred to the health centre.

### Statistical analysis

The main outcome was the incidence of clinical malaria during the follow-up period for a pair-wise comparison between any of the intervention arms i.e. SMC-AZc or SMC-APd *versus* control arm (SMC-A). Clinical malaria was defined as any case of medical issue that led to a consultation at the health centre and for which malaria was diagnosed after confirmation by RDT. Clinical malaria was either uncomplicated or severe regarding the absence of presence of severity criteria as defined by the national guidelines for malaria cases management. Secondary outcomes included RDT or microscopy positivity rates during the monthly home visits which were considered as asymptomatic cross-sectional parasitaemia. Participants in which no outcome was recorded and who missed at least 3 consecutive scheduled visits were considered as loss to follow-up.

Data were captured using eCRF designed on REDCap software and analysed with Stata 14 software (StataCorp). Statistical analysis was undertaken as outlined in the detailed study protocol published previously [25]. To this end, continuous and normally distributed data and categorical variables were described using mean (standard deviation) and proportions, respectively. For non-normally data, the median (Q25 and Q75) was used.

The effect of the intervention on uncomplicated malaria and on all-cause morbidity was assessed through the diagnosis at each attendance to health facilities (unscheduled visits), while asymptomatic infections were detected during monthly home visits using negative binomial regression, with results expressed as incidence risk ratio (IRR). For this, baseline adjustments were made for age, gender and the use of insecticide-treated bed nets, as they generally improve the precision of the treatment contrasts.

The risk of severe malaria was investigated using logistic regression. Similar predictors were used, as aforementioned. Variables with a P < 0.20 in univariate analyses were included in multivariable analyses.

## Results

**Fig. 1:**
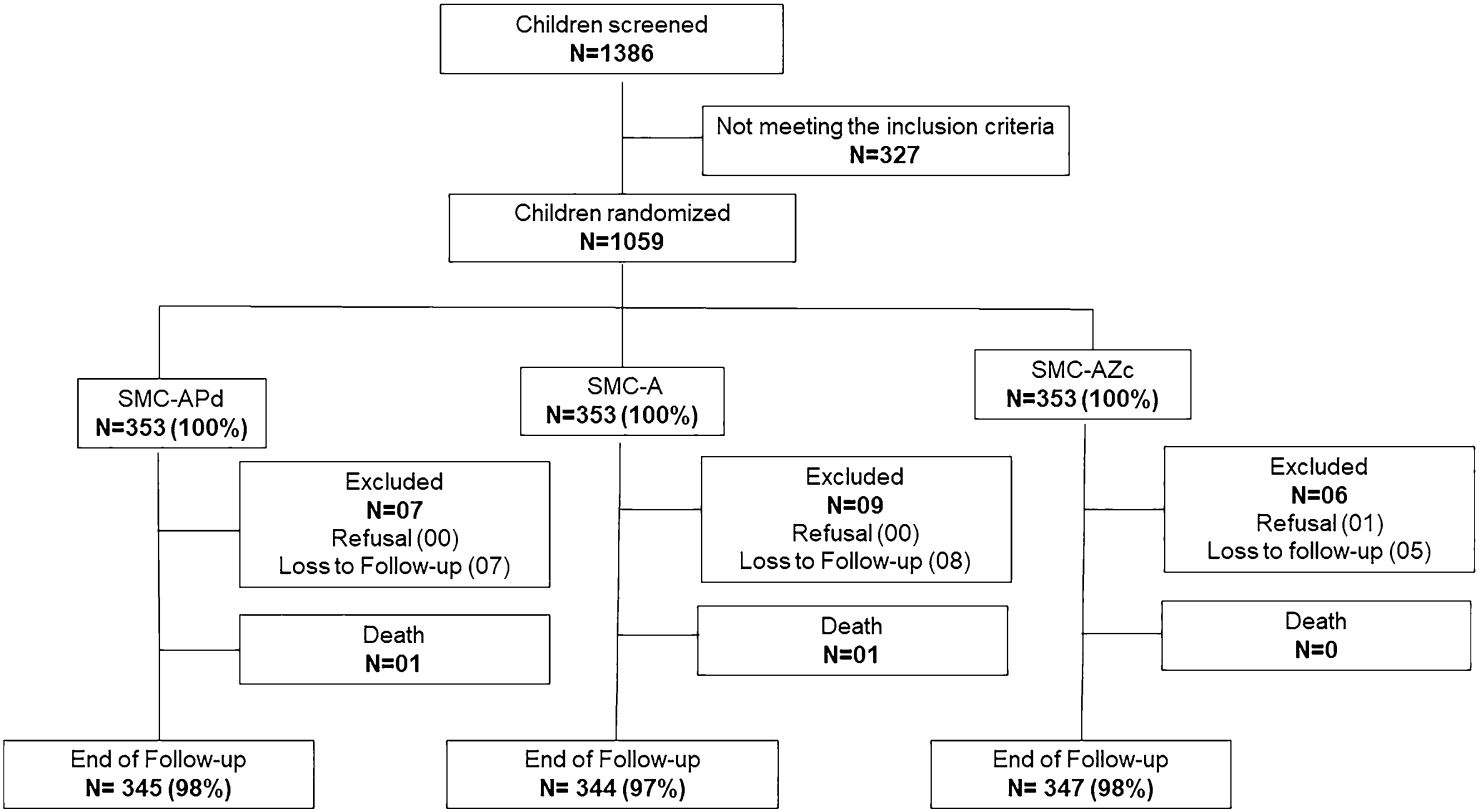
Trial profile. Flowchart of the study participant from inclusion to the end of the follow-up period

In accordance with the protocol, 1059 children were enrolled at a rate of 353 per arm. Out of them, 795 children were recruited in the area covered by Soaw medical centre, while 264 children were from area covered by the health and social promotion centre of Zoetgomdé. Figure 1 provides the flowchart of the participants from enrolment to the end of the follow-up period (Fig. 1). The baseline anthropometric parameters and malariological indices are presented in Table 1.

**Table 1:** Baseline socio-anthropometric characteristics and malarial profile of children

Characteristics	SMC-AZc n = 353	SMC-A n = 353	SMC-APd n = 353
Gender			
Male	170 (48.16%)	175 (49.58%)	185 (52.41%)
Female	183 (51.84%)	178 (50.42%)	168 (47.59%)
Age (months) Median (Q25–Q75)	35 (24 – 42)	36 (24 – 46.8)	34.8 (24 – 45.6)
RDT results			
Positive	79 (22.44)	70 (20.06)	85 (24.22)
Negative	273 (77.56)	279 (79.94)	266 (75.78)
Microscopy results			
Positive	42 (11.90)	27 (7.65)	40 (11.33)
Negative	311 (88.10)	326 (92.35)	313 (88.67)
Bed net use			
Yes	310 (88.32)	322 (91.22)	330 (93.48)
No	41 (11.68)	31 (8.78)	23 (6.52)
Mean Body temperature (°C)	35.87 (0.56)	35.89 (0.60)	35.86 (0.55)
Hb level (g/dL) Mean (SD)	10.09 (1.41)	9.96 (1.41)	10.01 (1.42)
Gametocyte carriage n (%)	7 (1.98)	10 (2.83)	8 (2.27)

After in-depth interview with the parents and verification of document, some children (n = 5 i.e. 3 in SMC-A arm, one in SMC-AZc arm and one in SMC-APd arm) were over the age of 59 months but were considered eligible because they were still included in the SMC target according to information from the health centres in each locality and were actually covered by the SMC campaign.

### Effect of nutrient supplementation on the incidence of uncomplicated malaria

**Fig. 2:**
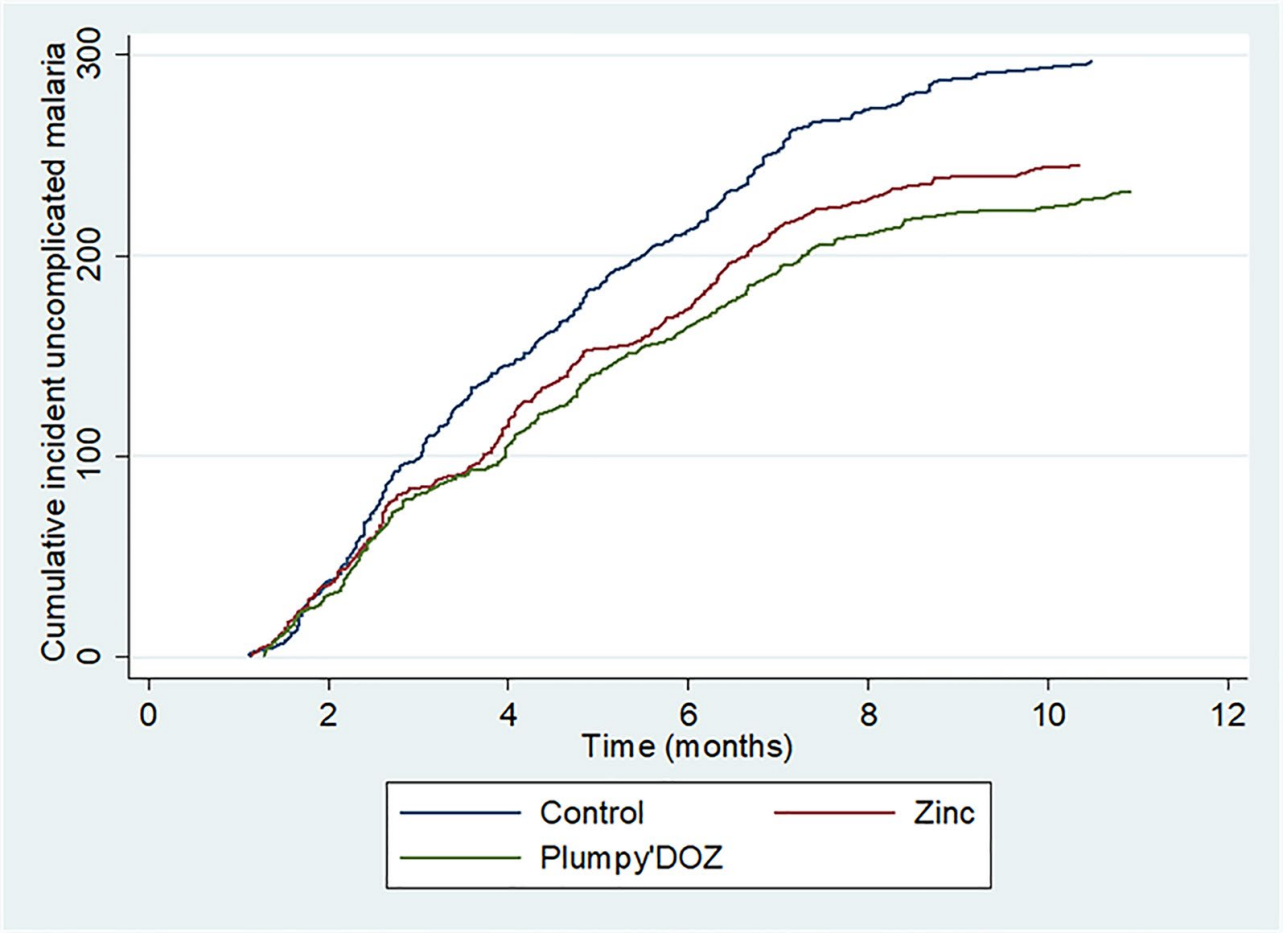
Cumulative incidence of uncomplicated malaria. Number of admissions to the health facilities due to uncomplicated malaria diagnosed clinically and confirmed by RDT per study arm form the beginning of the intervention to the end of the follow-up period. Note that each participant can contribute more than one episode of uncomplicated malaria over the study period. Blue curve represents control arm while green and red curves represent Plumpy’Doz and Zinc arm, respectively

From the start of the intervention until the end of the follow-up, 774 uncomplicated malaria cases were diagnosed in 1037 children. A total of 297 cases were observed in SMC-A arm while 245 and 232 cases were observed in SMC-AZc and SMC-APd arms, respectively. A representation of the temporal dynamic of uncomplicated malaria cases was provided in Fig. 2.

A reduction of 18% and 23% in the risk of having uncomplicated malaria was observed in SMC-AZc and SMC-APd arms respectively compared to the control arm (Table 2).

**Table 2:** Effect of the intervention on the incidence of uncomplicated malaria

Characteristics	Risk factor	Unadjusted		Adjusted	
		IRR (95%CI)	P-value	IRR (95%CI)	P-value
Sex	Male	1		1	
	Female	0.85 (0.70–1.03)	0.110	0.91 (0.75–1.10)	0.360
Age in years		0.72 (0.66–0.78)	< 0.001	0.72 (0.66–0.79)	< 0.001
ITN use	Yes	1		1	
	No	0.70 (0.48–1.03)	0.071	0.74 (0.49–1.12)	0.163
Intervention	SMC-A	1		1	
	SMC-AZc	0.82 (0.64–1.03)	0.098	0.82 (0.65–1.04)	0.094
	SMC-APd	0.77 (0.61–0.97)	0.033	0.77 (0.61–0.97)	0.031

### Effect of nutrient supplementation on the incidence of severe malaria

Throughout the follow-up period, 60 (25 in SMC-A arm, 23 in SMC-AZc and 12 in SMC-APd arm) cases of severe malaria were observed. Almost all cases resulted in severe anaemia (low Hb level). Except one fatal case which occurred in SMC-A arm (the second fatal case occurred in SMC-APd arm was not a malaria related case), the rest of severe malaria cases were successfully managed until recovery. A reduction of 52%, adjusted OR = 0.48 (0.23–0.98) in the risk of having severe malaria was observed in SMC-APd arm compared to control arm (Table 3).

**Table 3:** Effect of intervention on the risk of severe malaria

Characteristics	Risk factor	Unadjusted		Adjusted	
		OR (95%CI)	P-value	OR (95%CI)	P-value
Sex	Male	1			
	Female	0.97 (0.57–1.66)	0.921		
Age in years		0.74 (0.57–0.96)	0.025	0.74 (0.57–0.96)	0.027
ITN use	Yes	1		1	
	No	0.36 (0.08–1.51)	0.164	0.34 (0.08–1.44)	0.145
Intervention	SMC-A	1		1	
	SMC-AZc	0.94 (0.51–1.72)	0.854	0.94 (0.51–1.73)	0.854
	SMC-APd	0.50 (0.24–1.02)	0.060	0.48 (0.23–0.98)	0.045

### Effect of the intervention on asymptomatic carriage

**Fig. 3:**
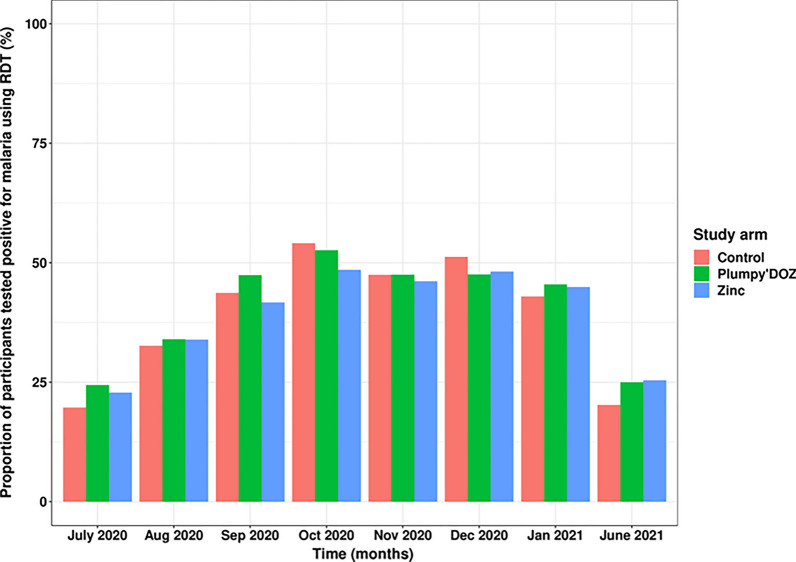
Cross-sectional RDT positivity rate per study arm. This figure displays the proportion of participants tested positive for malaria when deploying the RDT. For this, monthly visits data were used to estimate the proportions of infected participants to malaria when using RDT per study arm. Blue curve represents control arm while green and red curves represent Plumpy’Doz and Zinc arm, respectively

RDT positivity rate (RPR) during monthly home visits was similar in the three study arms. RPR was higher in October regardless the study arm. Unlike clinical episodes, no effect of nutrient supplementation on cross sectional asymptomatic infections was observed. Figure 3 represents the temporal dynamic of RPR throughout the follow-up period (Fig. 3). However, the figure shows a stretching of the high transmission peak towards the last quarter of the year. More specifically, infections were very frequent during the month of November and December suggesting the need for an extension of SMC during this period in the area.

### Effect of nutrient supplementation on all cause morbidity

**Fig. 4:**
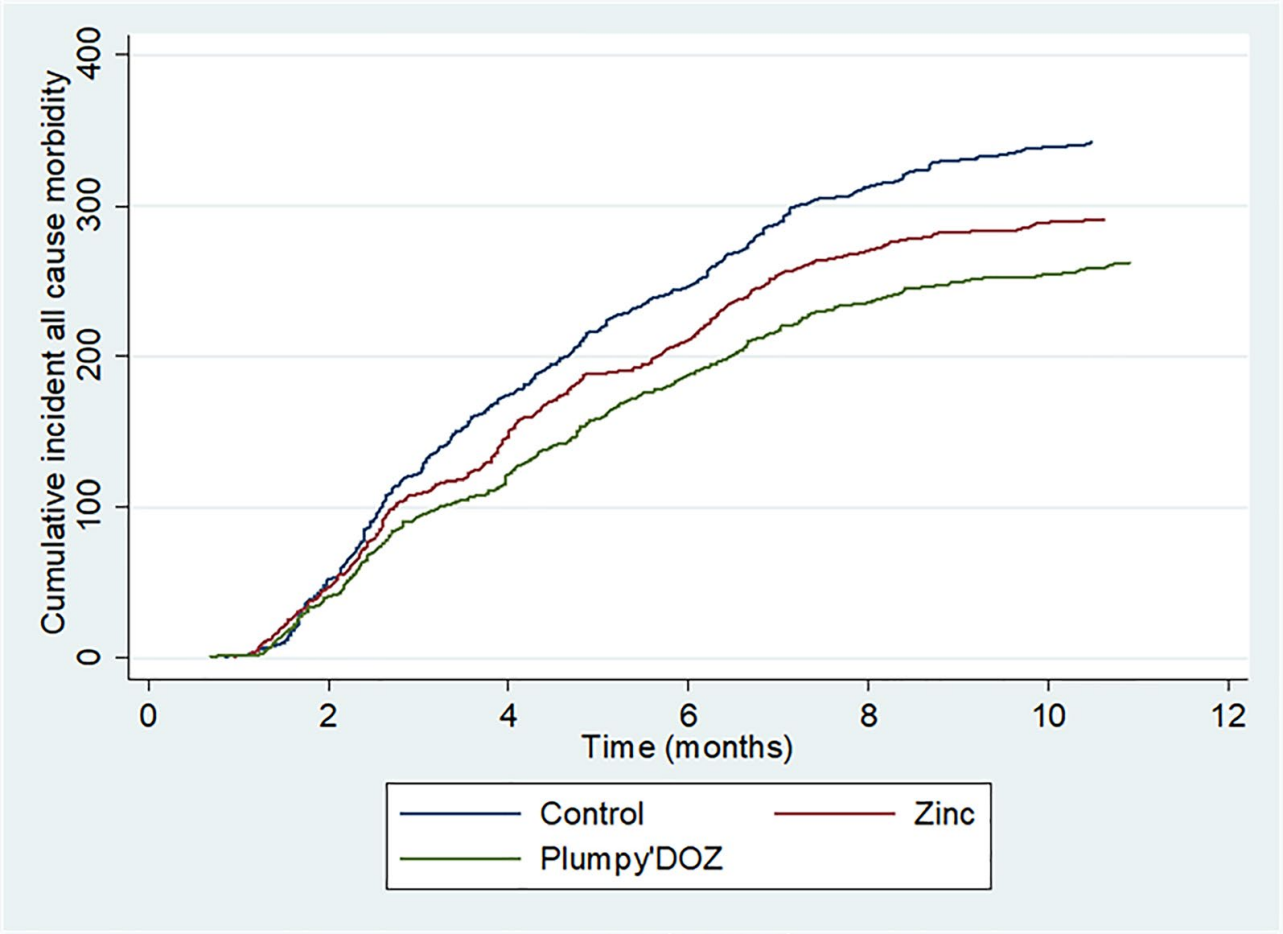
Cumulative incidence of all cause morbidity over the study period. Number of admissions to the health facilities due to a disease regardless the type of infections per study arm form the beginning of the intervention to the end of the follow-up period. Blue curve represents control arm while green and red curves represent Plumpy’Doz and Zinc arm, respectively. Note that each participant can contribute more than one episode of all cause morbidity over the study period

Disease burden was measured through the number of attendances to healthcare facilities from the start of the supplementation until the end of the study home visit regardless the type of infection. Overall, 895 attendances to healthcare facilities were observed. Out of them, 342 were registered in SMC-A *versus* 291 in SMC-AZc and 262 in SMC-APd. Figure 4 represent the cumulative incident of all cause morbidity over the study period (Fig. 4).

All cause morbidity was reduced in children receiving nutrient supplementation compared to control group. This reduction was more important and significant with children receiving Plumpy’Doz™ supplementation (Table 4).

**Table 4:** Effect of intervention on the incidence of all causes morbidity

Characteristics	Risk factor	Unadjusted		Adjusted	
		IRR (95%CI)	P-value	IRR (95%CI)	P-value
Sex	Male	1		1	
	Female	0.86 (0.71–1.03)	0.112	0.92 (0.77–1.10)	0.396
Age in years		0.70 (0.65–0.76)	< 0.001	0.71 (0.65–0.77)	< 0.001
ITN use	Yes	1		1	
	No	0.65 (0.44–0.95)	0.026	0.68 (0.46–1.01)	0.062
Intervention	SMC-A	1		1	
	SMC-AZc	0.84 (0.67–1.05)	0.140	0.84 (0.67–1.04)	0.120
	SMC-APd	0.76 (0.61–0.95)	0.019	0.75 (0.60–0.94)	0.014

## Discussion

Currently, there is a growing interest of combining health interventions for efficient management of resources to ensure sustainability while improving health outcomes [26]. This study demonstrated the feasibility of packaging malaria and nutritional intervention in sub-Saharan Africa.

Though previous studies reported the protective effect of AZc supplementation [19], no significant difference was observed either on all cause morbidity or malaria specific outcomes.

Interestingly, a synergetic action was observed with the MQ-LNS Plumpy’Doz™ enhancing the protective effect of SMC against clinical malaria while no effect on asymptomatic carriage was observed. This would mean that the addition of nutritional supplements would not protect the infection but would contribute to preventing the evolution of asymptomatic cases to clinical forms. Two plausible reasons initially evoked which could justify this result resides on the one hand in the reinforcement of the immune system by the nutritional supplements and on the other hand in the improvement of the absorption of the drug AQSP given during SMC campaign. The latter case seems less likely due to the lack of difference in asymptomatic carriage because increased drug absorption in children who received supplementation would necessarily lead to a difference not only in clinical cases but also in asymptomatic carriage. The persistence of the HRP2 protein in the absence of the parasite [27, 28] can influence this result, which constitutes a limit for this study based on RDT rather than microscopy, but the observation of the same trend over a whole year would reduce the effect of this bias resulting from the persistence of the HRP2 protein. More interestingly, the effect on all cause morbidity suggests a non-malaria specific effect and could support the immune enhancement hypothesis.

Beyond investigating the mechanisms underlying this synergistic effect, the most important relies in the extend of the reduction of the incidence of malaria as well as all cause morbidity by this combined strategy. In view of the level of reduction in the incidence of clinical malaria, especially severe malaria, this study demonstrates the need for this combined strategy in malaria control in endemic settings. Similarly, previous study conducted in Nigeria reported lower odds of clinical malaria in children who received a MQ-LNS in addition to SMC compared to children receiving SMC alone [22].

In terms of implementation, the door-to-door strategy for SMC offered an excellent opportunity for nutritional supplementation, which effectively reduces costs for implementation at scale. Acceptability of this combined strategy was not assessed, which represents a limitation for this study, but MQ-LNS are usually liked by children and their addition could also contribute to improving adherence to SMC. Another limit of this study relies in the duration of the supplementation, which was 3 months (delay in the acquisition of the Plumpy’Doz™ due to the closing of the borders during the Covid 19 pandemic) not covering the entire period of the SMC, which may reduce the expected effect.

Finally, the cross-sectional home visits during the follow-up allowed smooth characterization of the high transmission peak which was stretched towards the end of the year suggesting the need for an extension of SMC during this period in the area.

## Conclusion

Adding nutritional supplements to SMC significantly increases the impact of this intervention for preventing children from malaria and other childhood infections.

## Data Availability

Data supporting the conclusions of this article are included within the article and its additional files.

## Abbreviations

AQSP: Amodiaquine and sulfadoxine-pyrimethamine
HDSS: Health and Demographic Surveillance System
HRP2: Histidine rich protein 2
Pd: Plumpy’Doz
RDT: Rapid diagnosis test
RPR: RDT positivity rate
SMC: Seasonal malaria
Zc: Zinc chemoprevention
